# Circadian regulation of a limited set of conserved microRNAs in *Drosophila*

**DOI:** 10.1186/1471-2164-9-83

**Published:** 2008-02-19

**Authors:** Maocheng Yang, Jung-Eun Lee, Richard W Padgett, Isaac Edery

**Affiliations:** 1 Department of Molecular Biology and Biochemistry, Rutgers University, Piscataway, New Jersey, USA; 2 Waksman Institute, Rutgers University, Piscataway, New Jersey, USA; 3 Center for Advanced Biotechnology, Piscataway, New Jersey, USA; 4 National Cancer Institute, National Institutes of Health, Bethesda, Maryland, USA

## Abstract

**Background:**

MicroRNAs (miRNAs) are short non-coding RNA molecules that target mRNAs to control gene expression by attenuating the translational efficiency and stability of transcripts. They are found in a wide variety of organisms, from plants to insects and humans. Here, we use *Drosophila *to investigate the possibility that circadian clocks regulate the expression of miRNAs.

**Results:**

We used a microarray platform to survey the daily levels of *D. melanogaster *miRNAs in adult heads of wildtype flies and the arrhythmic clock mutant *cyc*^01^. We find two miRNAs (dme-miR-263a and -263b) that exhibit robust daily changes in abundance in wildtype flies that are abolished in the *cyc*^01 ^mutant. dme-miR-263a and -263b reach trough levels during the daytime, peak during the night and their levels are constitutively elevated in *cyc*^01 ^flies. A similar pattern of cycling is also observed in complete darkness, further supporting circadian regulation. In addition, we identified several miRNAs that appear to be constitutively expressed but nevertheless differ in overall daily levels between control and *cyc*^01 ^flies.

**Conclusion:**

The circadian clock regulates miRNA expression in *Drosophila*, although this appears to be highly restricted to a small number of miRNAs. A common mechanism likely underlies daily changes in the levels of dme-miR-263a and -263b. Our results suggest that cycling miRNAs contribute to daily changes in mRNA and/or protein levels in *Drosophila*. Intriguingly, the mature forms of dme-miR-263a and -263b are very similar in sequence to several miRNAs recently shown to be under circadian regulation in the mouse retina, suggesting conserved functions.

## Background

Circadian rhythms (approx. 24 hr) are governed by cellular "clocks" or pacemakers that can be synchronized to the daily and seasonal changes in external time cues (zeitgebers), most notably visible light and ambient temperature (reviewed in, [1]). This endogenously driven timing system enables life forms to anticipate environmental transitions, perform activities at biologically advantageous times during the day and undergo characteristic seasonal responses. An important challenge is to gain a better understanding of the biochemical and cellular bases underlying clocks.

Despite the wide variety of overt rhythms manifested by different species (e.g., from flowering in plants to wake-sleep cycles in humans), there is remarkable similarity in the underlying timing mechanisms. A core feature is based on species or tissue specific sets of 'clock' genes, whose RNA and protein products participate in interconnected positively and negatively acting transcriptional-translational feedback loops [2,3]. As a result of the design principles inherent in these autoregulatory molecular loops, one or more of the core clock RNA and protein products manifest 'self-sustained' daily rhythms in abundance that are instrumental for normal clock progression. The central clock transcription factors that participate in their own cyclical expression and that of other core clock genes also either directly or indirectly drive daily oscillations in the expression of many downstream effector genes. Microarray profiling studies indicate that ~1–10% of the genes expressed in a particular animal cell type undergo circadian fluctuations, demonstrating the strong influence of clocks in global gene expression [4-6]. Indeed, malfunctions in mammalian clock function have been linked to many diseases including cancer, metabolic syndromes and affective disorders [7-9].

Our understanding of clock mechanisms in general and mammalian ones in particular have been strongly based on findings using *Drosophila melanogaster *as a model system [1]. Key components of the intracellular clock mechanism in this species include PERIOD (*Drosophila *PER; dPER), TIMELESS (TIM), CLOCK (CLK) and CYCLE (CYC). CLK and CYC are transcription factors of the basic-helix-loop-helix (bHLH)/PAS (Per-Arnt-Sim) superfamily that heterodimerize to stimulate the daily transcription of *dper *and *tim*, in addition to other clock and downstream genes. After a time delay, dPER and TIM enter the nucleus where the association of dPER with the CLK-CYC heterodimer leads to the inhibition of CLK-dependent transcriptional activity. After several hours the levels of TIM and dPER undergo sharp decreases, relieving autoinhibition and initiating another round of CLK-CYC-dependent transcription. Mutations that either inactivate or severely impair the activities of *dper *(e.g., *per*^01^), *tim *(e.g., *tim*^0^) *cyc *(e.g., *cyc*^01^), or *Clk *(e.g., *Clk*^Jrk^), abolish molecular and behavioural rhythms [10].

MicroRNAs (miRNAs) are single-stranded RNA species of ~22 nucleotides that are derived by processing from a larger precursor and are found in a wide variety of organisms, from plants to insects and humans (recently reviewed in [11]). In animals, miRNAs usually have imperfect complementarity to elements in the 3' untranslated regions (UTRs) of mRNA targets, leading to post-transcriptional regulation of the target mRNA by inhibition of translation and/or stimulation of degradation [12-14]. It is thought that miRNAs mainly function to fine-tune the levels of key proteins. At present, estimates suggest there are about 100 miRNA encoding genes in insects, and over 400 in mammals. Although a comprehensive list of mRNAs targeted by miRNAs is still lacking in any organism, computational analysis suggests that a single miRNA could target 100s of genes [15]. Recent studies indicate that miRNAs have diverse physiological roles, such as apoptosis, homeobox regulation, viral infection, and development [11,16]. *Drosophila *has provided a powerful genetic and genomic model system to understand the biological roles of miRNAs [17,18]. Herein, we show that several miRNAs in *Drosophila *are clock-regulated, raising the possibility that they play fundamental roles in circadian systems.

## Results

### A limited number of miRNAs exhibit circadian regulation in Drosophila heads

**Figure 1:**
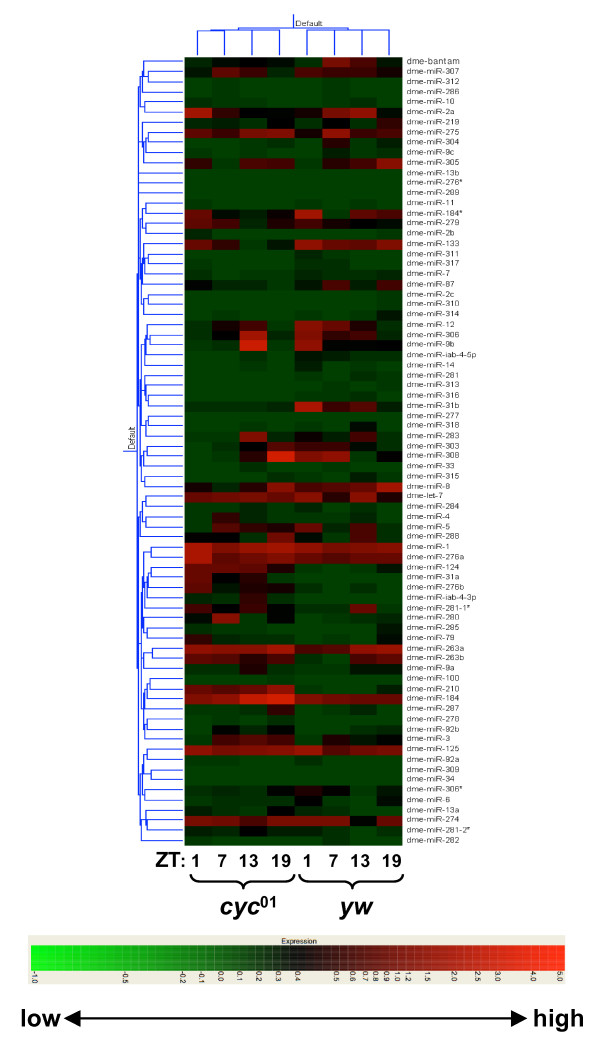
**Heatmap of *Drosophila *miRNA expression as a function of daily time and in *cyc*^01 ^flies**. Colorgram depicts the relative levels of miRNAs (high, red; average, black; low, green--as indicated by color bar, bottom) and summarizes hierarchical clustering of daily light-dark patterns of miRNAs in control (*y w*) and mutant (*y w*;;*cyc*^01^) flies using Genespring GX (Agilent, Santa Clara, CA). Flies were collected at ZT1, 7, 13 and 19, as indicated (bottom of panel).

To determine whether miRNAs exhibit daily changes in abundance we entrained control *D. melanogaster *flies (*y w*) to standard 12 hr light/12 hr dark cycles [12:12LD; where zeitgeber time 0 (ZT0) is defined as beginning of the light phase] at 25°C and collected flies at 6 hr intervals during the third LD cycle (i.e., ZT1, 7, 13 and 19). In addition, we analyzed the well-characterized *cyc*^01 ^arrhythmic mutant [19]. The wildtype control and mutant *cyc*^01 ^flies used here are in the same *y w *genetic background and were treated contemporaneously. RNA was prepared from adult heads, which contain the key pacemaker neurons driving rhythmic behavior [20] and are routinely used to evaluate cycling profiles of mRNAs in *Drosophila*[5,21]. We used the miRMAX microarray platform with the Array900 miRNA Direct Labeling System [22] that features dimer probes complementary to mature or abridged miRNA sequences of all the identified miRNAs of *Drosophila *according to the miRBase version 5.1 [23]. For each time point, three independent experiments were analyzed and data pooled (Fig. 1). The Gene Expression Omnibus (GEO) accession number for our microarray raw data is GSE10005.

To identify clock-regulated miRNAs we applied several criteria. First, there had to be at least a statistically significant difference between the lowest and highest signal intensity of the four time points analyzed in wildtype flies. We applied the stringent test of ANOVA with FDR (5%) to compare within each genotype as a function of time, and between the *y w *control and *y w*;;*cyc*^01 ^(herein, more simply refereed to as *cyc*^01^) mutant groups. Second, a *bona-fide *clock-regulated miRNA should not exhibit statistically significant difference between the four time points in *cyc*^01 ^flies; i.e., levels should remain constant throughout a daily cycle--as is observed for clock-controlled mRNAs in this mutant [19,24]. Finally, we required a minimum difference of 50% between peak and trough values in wildtype flies.

**Figure 2:**
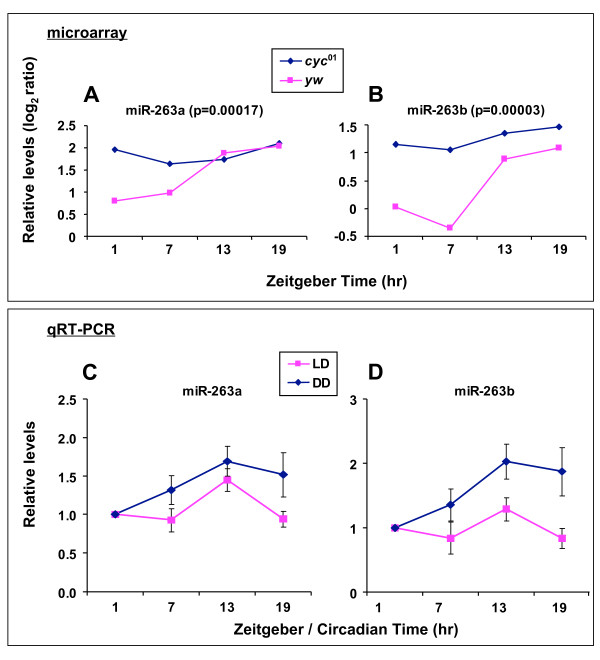
**MiRNAs showing significant differences in levels as a function of daily time within the *y w *control group**. **(A, B) ***Y w *or *y w*;;*cyc*^01 ^flies were collected at the indicated times during the third LD cycle. LMW RNA was prepared from head extracts and subjected to miRNA microarray profiling. Shown are the miRNAs with statistically different levels as a function of time within the *y w *group but not within the *y w*;;*cyc*^01 ^group. P values are indicated for each miRNA. **(C, D) ***Canton-S *flies were collected at the indicated times during either the third LD or the first day of DD. Total RNA was prepared from head extracts and subjected to quantitative RT-PCR (qRT-PCR). Results reflect the average of four replicates from two independent experiments. Error bars are S.E.M.

We obtained significant signals from 78 *Drosophila melanogster *miRNAs (Fig. 1). Of these, only two miRNAs (dme-miR-263a and -263b) in wildtype control flies exhibited daily abundance changes that were statistically significant using the ANOVA with 5% FDR test (Fig. 2A and 2B). Importantly, the levels of miR-263a and -263b were constant throughout a daily cycle in the *cyc*^01 ^mutant, indicative of *bona-fide *circadian regulation (Fig. 2A and 2B). Intriguingly, both miRNAs attain trough levels during the daytime hours and have constitutively elevated levels in the *cyc*^01 ^mutant, suggesting a common mechanism underlying their clock regulation (see Discussion). We confirmed that both dme-miR-263a and -263b cycle using quantitative RT-PCR (qRT-PCR) and also showed that these oscillations persist in constant dark conditions (Fig. 2C and 2D). Although it is not clear why the daily profiles are slightly different when comparing results obtained using the microarray platform or qRT-PCR, the combined results clearly indicate circadian regulation in the levels of dme-miR-263a and -263b.

**Figure 3:**
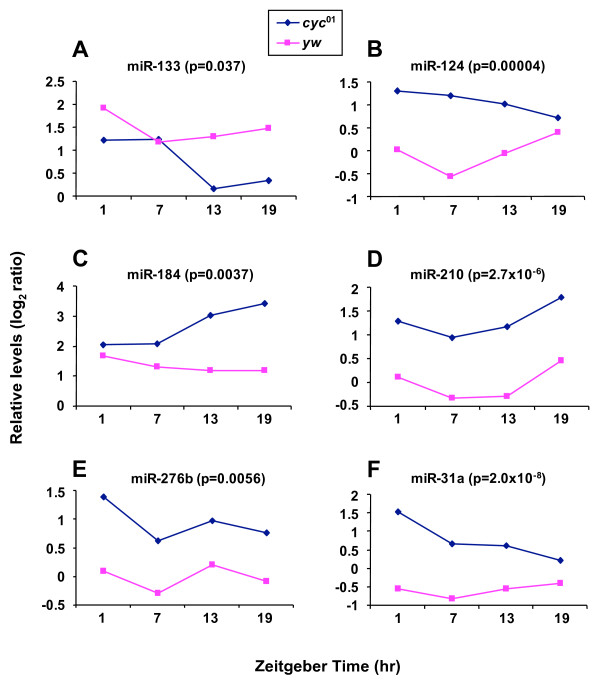
**MiRNAs showing significant differences in overall daily levels between *yw *and *cyc*^01 ^flies**.*Y w *or *y w*;;*cyc*^01 ^flies were collected at the indicated times during the third LD cycle. LMW RNA was prepared from head extracts and subjected to miRNA microarray profiling. Shown are the miRNAs with statistically different overall daily levels between the *y w *and *y w*;;*cyc*^01 ^groups. P values are indicated for each miRNA.

No miRNAs that we evaluated showed significant differences in levels as a function of time in *cyc*^01 ^flies, suggesting that (at least in the absence of a functional clock) the light-dark cycle has little to no direct effect on the expression of miRNAs in *Drosophila *heads. We also identified miRNAs that do not exhibit statistically significant changes throughout a daily cycle in wildtype flies but nonetheless show differences in average daily levels when compared to *cyc*^01 ^flies (Fig. 3). In most cases, the average daily values in *cyc*^01 ^flies were higher compared to control flies. Of these, dme-miR-124 showed a pattern very similar to that of dme-miR-263a and -263b, exhibiting trough levels during the mid-day that were followed by increases during the early to late-night in wildtype flies and constantly elevated levels in the *cyc*^0 ^mutant (Fig. 3B). Although the differences in daily levels for miR-124 in wildtype flies do not reach significance even when less stringent criteria was applied (p = 0.0813, ANOVA without FDR), it is possible that miR-124 undergoes low amplitude circadian oscillations in abundance. Likewise, miR-31a (Fig. 3F) might also exhibit low amplitude cycling similar to that of miR-124.

### Possible circadian-relevant targets of dme-miR-263a and 263b

Experimental and computational studies have shown that several hundred different mRNAs can be targeted by each miRNA [15,25]. As an initial attempt to identify possible targets for the miRNAs that either show circadian regulation or differences in overall daily levels between control and *cyc*^01 ^flies (Figs. 2 and 3) we used four readily available web-based prediction programs that contain information on *D. melanogaster *(i.e., PicTar [26], miRBase [23], TargetScan [27] and EMBL [28]).

Cycling mRNAs that were previously identified based on whole genome expression profiling [29-34] are found among the predicted targets of the clock-regulated miRNAs we detected in this study (data not shown). This is to be expected based simply on the many clock regulated genes reported to date, the possibility that a single miRNA can target many different transcripts and the use of multiple prediction programs. However, although hundreds of circadian regulated mRNAs have been identified in *Drosophila *using microarray-based strategies, there is little overlap between the different studies, except for the high-amplitude core clock transcripts [5,31,35]. In addition, miRNAs apparently function in animals by mainly regulating translational efficiency [13,14], suggesting that constitutively expressed mRNAs are equally good targets of miRNAs to generate daily fluctuations in protein levels. Thus, we limited our focus of possible targets to genes that function within the central clock mechanism or with characterized roles in the *Drosophila *circadian timing system (Table 1).

**Table 1:** Predicted clock-relevant targets for miRNAs identified in this study

**miRNA**	**Target^a^**
dme-miR-263a	*Clk *[T]^b^, *dbt *[P], *tws *[T], *slo *[S]
dme-miR-263b	*Clk *[T], *cwo *[P, E]
dme-miR-31a	-
dme-miR-124	*jet *[S]
dme-miR-133	*CkIIα *[S], *slo *[S]
dme-miR-184	-
dme-miR-210	*per *[S], *CkIIβ *[S]
dme-miR-276b	*tim *[S, T], *CkIIβ *[S]

^a^The predicted targets were limited to the following clock relevant genes (annotation symbol); *dper *(CG2647), *tim *(CG3234), *Clk *(CG7391), *cyc *(CG8727), *vri *(CG14029), *Pdp1 *(CG17888), *cwo *(CG17100), *dbt *(CG2048), *CkIIα *(CG17520), *CkIIβ *(CG15224), *sgg *(CG2621), *slmb *(CG3412), *tws *(CG6235), *wdb *(CG5643), *mts *(CG7109), *jet *(CG8873), *cry *(CG3772), *slo *(CG10693) and *pdf *(CG6496).

^b^The following abbreviations are used: P, Pictar; S, miRBase (Sanger); T, TargetScan; E, EMBL.

Using this more limited search, we noted that *Clk *might be a target of both miR-263a and -263b (Table 1). However, this was only predicted by TargetScan and might be an artefact of using a *Clk *sequence with a premature translation stop codon (i.e., the region in question lies upstream to the normal translation stop codon). In addition, although *Clk *RNA levels cycle [36], the total abundance of the protein is constant throughout a daily cycle [37,38]. Perhaps a more promising candidate is *clockwork orange *(*cwo*), which is a transcriptional repressor recently shown to modulate circadian rhythms in *Drosophila *[39-41]. Two independent search programs (Pictar and EMBL) predict that *cwo *might be targeted by miR-263b. Expression of *cwo *is directly driven by CLK-CYC and exhibits daily RNA cycles that peak in the early night with trough values attained during the late night/early day [39-41]. miR-263b has a similar RNA profile as *cwo*, raising the possibility that it functions to attenuate translation of *cwo *transcripts as they accumulate during the night.

Other possible targets of miR-263a and miR-263b include *doubletime *(*dbt*) and *twins *(*tws*), respectively. DBT is the *Drosophila *homolog of casein kinase 1ε (CK1ε), whereas TWS is a protein phosphatase type 2A regulator. Both play important but presumably opposing or balancing roles in the *Drosophila *circadian system by targeting key clock proteins, such as dPER and CLK (reviewed in, [42]). Prior work has shown that at least one isoform of the *tws *transcripts cycles in abundance, rising during the late day and decreasing during the night, while remaining constitutively low in the *cyc*^01 ^mutant [43]. Intriguingly, miR-263b expression is essentially anti-phase to that of the cycling *tws *transcript and is constantly high in *cyc*^01 ^flies (Fig. 2). Thus, miR-263b could amplify daily oscillations in the levels or translational efficiency of *tws *RNA. While speculation is tempting, just one out of the four prediction programs identified *dbt *and *tws*. Finally, the physiological significance of targeting *dbt *expression in a circadian manner by miRNAs is suspect because neither *dbt *mRNA nor protein levels cycle [44].

With regards to the other class of miRNAs that show differences in overall daily levels between wildtype and *cyc*^01 ^flies (Fig. 3), we noted several clock-relevant genes as putative targets (Table 1). A possibly noteworthy finding is that casein kinase 2 (CKII), which has a role in the *Drosophila *clock [45,46], is predicted to be a target of miR-133, miR-210 and miR-276b. It is thought that the presence of multiple miRNA interaction sites allows for exquisite control of target mRNA expression [47]. Nonetheless, only a single prediction program (in this case, Sanger) identified *CkII *as a potential target of these miRNAs. Besides *cwo*, the only clock gene that was predicted by more than one program as a potential target of a single miRNA was *tim*, a candidate target of miR-276b (Table 1). Clearly, future studies will be required to verify if any of the miRNAs identified in this study play roles in the clock and/or rhythmic expression.

## Discussion

Our results indicate that the levels of a limited number of miRNAs in *D. melanogaster *exhibit robust circadian regulation. Of the 78 miRNAs that we probed by expression profiling only dme-miR263a and -263b displayed strong evidence of circadian regulation (with possible weak cycling for dme-miR-124). Importantly, the daily cycles in dme-miR263a and -263b were abolished in the *cyc*^01 ^mutant, and persisted in constant dark conditions (Fig. 2). These results indicate that changes in the amounts of miR-263a and -263b are not merely driven by daily light-dark cycle but are dependent on a functional clock. Moreover, we did not detect other miRNAs that display robust cycling profiles in daily light-dark cycles, suggesting that the expression of miRNAs in *D. melanogaster *is largely insensitive to photic signals. However, we cannot rule out transient effects of light. In addition, it is possible that highly stable miRNAs, despite circadian regulation in expression, would not exhibit daily cycles in abundance. Also, as an initial foray into determining whether miRNA expression in *Drosophila *is under circadian regulation we used total head extracts as a source for miRNA isolation, which could mask cell or tissue specific temporal regulation. Despite these caveats the limited number of cycling miRNAs strongly suggests that these molecular rhythms mainly arise at the level of transcriptional regulation and not global oscillations in the miRNA-processing machinery.

At least two interconnected transcriptional feedback loops operate within the core clock mechanism in *Drosophila *[48]. In the 'major' loop, CLK and CYC form a heterodimer that binds E-box containing elements found in 5' regulatory regions of *dper*, *tim *and *vrille *(*vri*), in addition to numerous other downstream effector genes. While dPER and TIM cooperate to negatively regulate CLK-CYC transactivation, VRI functions in an interacting transcriptional loop by binding to 5' upstream regulatory elements on *Clk*, an event that inhibits *Clk *expression. As a result of the molecular logic underlying the intertwined transcriptional circuitry, *dper*, *tim *and *vri *mRNA levels follow a similar temporal profile peaking in the early night, whereas *Clk *transcripts exhibit essentially anti-phase cycling [36].

MiR-263a and -263b have similar daily profiles, with peak levels in the early to mid-night (between ZT/CT13 and ZT/CT19) and trough amounts reached during the early to mid-day (ZT/CT1-7) (Fig. 2). Moreover, both miRNAs are constitutively expressed at peak levels in the *cyc*^01 ^mutant, suggesting a common mechanism is controlling daily changes in the levels of miRs -263a and -263b. The daily patterns of changes in the levels of miR-263a and -263b are somewhat reminiscent of mRNA targets directly regulated by CLK-CYC. However, whereas the levels of *bona-fide *direct targets of CLK-CYC, such as *dper *and *tim*, are reduced in *cyc*^01 ^flies [19,24], the abundance of miR-263a and -263b are pegged at peak amounts (Fig. 2). These considerations raise the possibility that the circadian expression of miR-263a and -263b is not directly driven by core clock transcription factors. For example, CLK-CYC might stimulate the rhythmic expression of a transcriptional inhibitor that blocks expression of miR-263a and -263b during the day.

**Figure 4:**
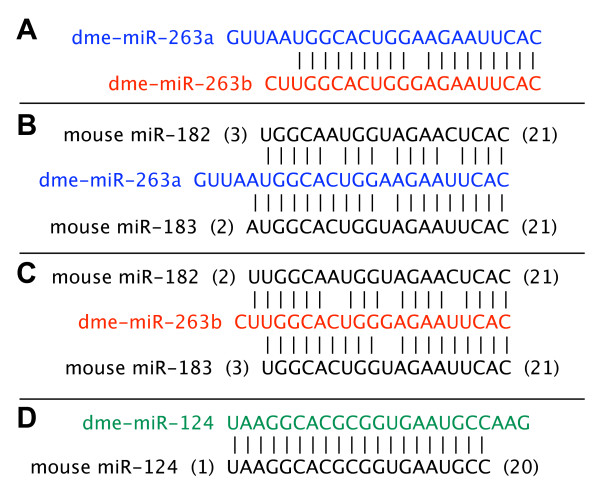
**Similarity between some of the miRNAs identified in this study and recently described miRNAs that cycle in mammals**. Shown are the alignments between miRNAs identified in this study and possible mammalian orthologs that were recently described by Xu et al. (2007) as cycling in the mouse retina. The entire sequences of the mature forms of *Drosophila *miRNA are shown in different colors, whereas mouse sequences are shown in black. The identity of the miRNA is shown at left; number in brackets signifies the nucleotide position of the mature mouse miRNA starting from left (5') to right (3'). Sequences used were those in the Sanger miRBase as of December 2007.

Besides targeting individual genes, clusters of genes can be co-ordinately regulated by the circadian clock in *Drosophila*, presumably as a result of temporal changes in chromatin remodelling [33]. In a somewhat analogous manner, recent evidence shows that multiple unique miRNAs can be generated and co-regulated from a single primary miRNA (pri-miRNA) transcript [49,50]. However, even though miR-263a and -263b are paralogous genes in the same family (miR-263) with very similar mature sequences (Fig. 4A), they are found on the second and third chromosomes, respectively. The fact that these two miRNAs have closely related target sites and undergo similar circadian regulation suggests that they have parallel functions in contributing to rhythms in the expression of an overlapping set of genes. Nonetheless, recent work comparing 12 *Drosophila *genomes suggest that some of the miRNA sequences currently in use need to be revised, which could result in marked changes in predicted targets [51]. This could be especially relevant to this study as evolutionary evidence suggests a different 5' end for miR-263a [51].

Although we also identified several (apparently) non-cycling miRNAs with statistically different overall daily levels between wildtype and *cyc*^01 ^mutants (Fig. 3), the physiological significance of these results is presently not clear. Nonetheless, whole genome microarray profiling in *Drosophila *has shown that mutations in core clock transcription factors not only abolishes rhythmic expression but can lead to changes in the basal levels of many constitutively expressed mRNAs [34]. Future work will be instrumental in identifying *in vivo *targets of the miRNAs identified in this study and evaluate their putative roles in the *Drosophila *circadian system.

While this manuscript was in preparation, evidence of miRNA cycling and roles in the clock were shown in mammals. One study identified miR-132 and miR-219 as being clock regulated in the suprachiasmatic nucleus (SCN), the 'master clock' in mammals [52]. In addition, they also showed that miR-132 plays a role in circadian photic entrainment, whereas miR-219 modulates period length. In another study, several miRNAs specifically expressed in the mouse retina exhibited daily oscillations in levels [50]. Among the subgroup of miRNAs showing circadian regulation in the retina were members of the miR-183/96/182 cluster. We used the miRBase database to determine if any of the miRNAs we identified are similar to the recently described cycling miRNAs in mammals. We used the mature sequences of the *D. melanogaster *miRNAs as queries to search the database for vertebrate miRNAs with similar sequences. Although potentially coincidental, the mature sequence for miR-183 in vertebrates is very similar to that of both *D. melanogaster *dme-miR-263a and miR-263b (Figs. 4B and 4C). The *D. melanogaster *dme-miR-263b is also similar to the vertebrate miR-182 (three mismatches over the length of 20 aligned nucleotides). Finally, the *D. melanogaster *dme-miR-124 is similar to vertebrate miR-124a (Fig. 4D), which also cycled in the mouse retina [50]. Because we probed whole heads where the majority of the clocks reside in the compound eyes [53], this might have contributed to the surprising overlap in cycling miRNAs between our study and those identified in the mouse retina. Of the miRNAs we identified only miR-276b appears not to be conserved in vertebrates. Thus, our results raise the interesting possibility that similar miRNAs have conserved functions in the circadian systems of vertebrates and insects, perhaps in a tissue-specific manner.

## Conclusion

A small proportion of miRNAs in *Drosophila *exhibit robust clock-regulated rhythms in abundance. Our results suggest that for some proteins, daily changes in their levels are modulated by miRNA-mediated post-transcriptional regulation. It is likely that the action of miRNAs in rhythmic expression is to provide a fine-tuning mechanism that coordinates with transcriptional and post-translational pathways to generate an oscillatory system that can adjust the levels of specific proteins to values that are appropriate for a given time of day.

## Methods

### Fly strains and treatment

*Drosophila *strains used were descendants of *Canton-S*, *yw *and *yw*;;*cyc*^01 ^strains previously described [19]. Flies were maintained in standard media at 25°C. For collections, approximately 30–100 young (2–5 day old) flies were placed in vials that were incubated at 25°C for three to four days in standard 12 hr light-12 hr dark cycles [LD; where zeitgeber time 0 (ZT0) is defined as lights-on], and in some cases followed by complete darkness [DD; where circadian time 0 (CT0) is defined as subjective lights-on]. Flies were collected by freezing in dry ice during the third or fourth LD cycle or the first DD cycle at the following times; ZT/CT1, 7, 13 and 19. Subsequently, heads were isolated from the frozen flies and kept at -70°C until further processed, as described below.

### RNA preparation and labelling

Adult fly heads were flash frozen and ground into fine powder in liquid nitrogen for miRNA microarray analysis. Low molecular weight (LMW) RNA was purified using the mirVana™ miRNA extraction kit (Ambion, Austin, TX, USA). About 250 ng of [23]LMW RNA was used as the input for miRNAs labelling using the Array900 miRNA Direct kit (Genisphere Inc., Hatfield, PA, USA), a tagging method that allows direct labeling of mature miRNAs without PCR amplification. All samples were labelled with Cy5 dye. A pooled control was labelled with Cy3 and equal amount of the tagged Cy3 was hybridized to every chip. The fluorescent (Cy5/Cy3) signal of each labelled miRNA was amplified about 850–900 times after being tagged with 3DNA dendromer in hybridization [22].

### MiRNA microarrays

For microRNA expression evaluation, we used miRMAX microarray technology [22] that features tandem dimer probes complementary to mature microRNA sequences (or abridged sequence) of all the identified miRNAs of *Drosophila *according to the miRNA registry VERSION 5.1 [23]. The total number of miRNAs on the chip is 1231, which includes 1087 miRNAs from miRbase 5.1 and 144 predicted human miRNAs. The chip contains several subarrays for all miRNAs from *Drosophila*, *C. elegans*, human, mouse, and rat so it can be used for probing various organisms. We only extracted data from the *Drosophila *subarray. The miRMAX microarray can detect miRNA expression with only 100 ng LMW RNA input. Three independent RNA samples for each time point were hybridized and analyzed. Microarray chips were hybridized in Hybex Microarray Incubation System (SciGene, Sunnyvale, CA) and processed according to the protocol of the Array900 miRNA Direct kit (Genisphere Inc., Hatfield, PA, USA). A GenePix 4000B scanner (Axon Instruments, Union City, CA, USA) was used to scan individual chips and median spot intensities were generated using GenePix 4.0 (Axon Instruments). The Gene Expression Omnibus (GEO) accession number for our microarray raw data is GSE10005.

### Statistical analysis for microarrays

Scanned microarray raw data were processed and normalized using a GeneTraffic Duo server on campus (Strategene, La Jolla, CA, USA). We used the Cy3 labelled *cyc*^01 ^ZT1 samples (About 200 ng LMW RNA input for each chip) for normalization, so that we could compare ratios of a given miRNA relative to the same sample. Data were transformed into log2 ratio and were further analyzed by using the Genespring GX software (Agilent, Santa Clara, CA). First, miRNAs from all genes were tested with Welch ANOVA among the following groups based on time points ZT1, 7, 13, and 19 in both *yw *and *cyc*^01 ^origin; p-value cutoff 0.05, multiple testing correction: Benjamini and Hochberg False Discovery Rate (FDR). Then, Welch t-test was used to identify significantly differentially expressed miRNAs between "origin *y w *" and "origin *cyc*^01 ^" from the Volcano Plot built. Multiple Testing Correction: Benjamini and Hochberg False Discovery Rate. Which miRNAs were differentially expressed was defined by Fold Difference, 1.5 and a P-value Cutoff, 0.05.

### Quantitative RT-PCR

Approximately 30 to 40 adult flies were frozen on dry ice at the indicated times during either LD or DD and kept at -80°C until further processing. Fly heads were isolated and crushed in 200 μl of TRI Reagent (Sigma) with a motorized pestle, as previously described [54,55]. Total RNA was prepared according to the manufacturer's instruction and the final RNA pellet was resuspended in 12–15 μl of DEPC-treated water. For the analysis of miR-263a and miR-263b, 4 μl of total RNA solution were subjected to reverse transcription in the presence of miRNA-specific primers as supplied by the manufacturer (TaqMan MicroRNA Assay, Applied Biosystems). The resulting cDNAs were diluted 15-fold in 1 mM Tris-HCl (pH 8.0). Subsequently, 2 μl of cDNA solution were added to a total of 20 μl and PCR reactions performed in triplicate using either the miR-263a or miR-263b specific primers, according to the manufacturer's instructions (TaqMan MicroRNA Assay, Applied Biosystems). We used oligo-dT primed cDNA synthesis to measure the levels of the internal controls, *rp49 *or *cbp20*, as previously described [54,55]. Briefly, 2 μl of total RNA solution was used for reverse transcription (Omniscript RT, Qiagen). The resulting cDNAs were diluted 10-fold in 1 mM Tris-HCl (pH 8.0). Subsequently, 2 μl of cDNA solution were added to a total of 30 μl and PCR reactions performed in triplicate (QuantiTect SYBR Green PCR, Qiagen) with the following conditions: an initial step of 15 min at 95°C to activate HotStarTaq DNA polymerase, followed by 40 cycles of 15 s at 94°C, 30 s at 60°C, and 30 s at 72°C. All PCR reactions were performed in 96-well plates using an ABI prism 7600 system (Applied Biosystems). Standard curves were generated with serially diluted cDNA samples for every run and the relative copy number of the gene of interest was calculated based on Ct (threshold cycle) values. The values for miR-263a and miR-263b were normalized to the relative copy number of *rp49 *or *cbp20 *cDNAs. Finally, the relative levels of miR-263a and miR-263b at ZT/CT1 were set to 1.0 and the other values normalized. The efficiency of all the PCR reactions was at least 90%.

## Authors' contributions

MY prepared the RNA samples for microarray analysis, did the microarrays and performed the statistical analysis. JL collected the flies, helped in the RNA preparation and also did the quantitative RT-PCR experiments. IE and RWP conceived of the study, supervised the work and contributed to writing the manuscript. All authors read and approved the final manuscript.
